# Growth differentiation factor-15 and fibroblast growth factor-23 are associated with mortality in type 2 diabetes – An observational follow-up study

**DOI:** 10.1371/journal.pone.0196634

**Published:** 2018-04-26

**Authors:** Marie Frimodt-Møller, Bernt Johan von Scholten, Henrik Reinhard, Peter Karl Jacobsen, Tine Willum Hansen, Frederik Ivar Persson, Hans-Henrik Parving, Peter Rossing

**Affiliations:** 1 Steno Diabetes Center Copenhagen, Copenhagen, Denmark; 2 Department of Cardiology, Rigshospitalet, Copenhagen University Hospital, Copenhagen, Denmark; 3 Department of Medical Endocrinology, Rigshospitalet, Copenhagen University Hospital, Copenhagen, Denmark; 4 University of Copenhagen, Copenhagen, Denmark; University of Glasgow, UNITED KINGDOM

## Abstract

**Objectives:**

Two biomarkers, growth differentiation factor 15 (GDF-15) and fibroblast growth factor 23 (FGF-23)), reflecting different aspects of renal pathophysiology, were evaluated as determinants of decline in estimated glomerular filtration rate (eGFR), incident cardiovascular disease (CVD) and all-cause mortality in patients with type 2 diabetes (T2D) and microalbuminuria, but without clinical cardiac disease.

**Materials and methods:**

Prospective study including 200 T2D patients. The predefined endpoint of chronic kidney disease (CKD) progression: A decline in eGFR of >30% at any time point during follow-up. Hazard ratios (HR) are provided per 1 SD increment of log2-transformed values.

**Results:**

Mean (± SD) age was 59 ± 9 years, eGFR 91.1 ± 18.3 ml/min/1.73m^2^ and median (IQR) UAER 103 (39–230) mg/24-h. During a median 6.1 years follow-up, 40 incident CVD events, 26 deaths and 42 patients reached the CKD endpoint after median 4.9 years. Higher GDF-15 was a determinant of decline in eGFR >30% and all-cause mortality in adjusted models (HR 1.7 (1.1–2.5); p = 0.018 and HR 1.9 (1.2–2.9); p = 0.003, respectively). Adding GDF-15 to traditional risk factors improved risk prediction of decline in renal function (relative integrated discrimination improvement (rIDI) = 30%; p = 0.037). Higher FGF-23 was associated with all-cause mortality in adjusted models (HR 1.6 (1.1–2.2); p = 0.011) with a rIDI of 30% (p = 0.024).

**Conclusions:**

In patients with T2D and microalbuminuria, higher GDF-15 and FGF-23 were independently associated with all-cause mortality and higher GDF-15 improved risk prediction of decline in kidney function and higher FGF-23 of all-cause mortality, beyond traditional risk factors, but not independently of GDF-15.

## Introduction

Patients with type 2 diabetes (T2D) are at significantly increased risk for both cardiovascular disease (CVD) and development of chronic kidney disease (CKD)[1]. Diabetic kidney disease is the most frequent cause of end-stage renal disease (ESRD) in western countries[2]. Moreover, progression of CKD further increases the risk for CVD[3].

Identification of persons at highest risk of rapid progression in CKD would allow stratification in an early phase so adequate treatment can be implemented. Currently, presence of albuminuria and lower estimated glomerular filtration rate (eGFR) are the best means to identify persons at highest risk of ESRD[4]. However, the search for additional biomarkers to improve this stratification is ongoing.

Growth differentiating factor (GDF)-15, a member of the transforming growth factor beta (TGF-β) cytokine superfamily and the bone-derived hormone fibroblast growth factor (FGF)-23 are two biomarkers reflecting different aspects of renal pathophysiology. GDF-15 is considered as a marker of inflammation and oxidative stress and FGF-23 as a regulator of the phosphate homeostasis, vitamin D metabolism and bone mineralization. Both biomarkers have been suggested as risk markers of cardiovascular disease, mortality and decline in renal function in the general population[5–8], and in patients with CKD[9–15] or CVD[16]. There are however conflicting results regarding GDF-15 and FGF-23 as predictors of these outcomes in patients with T2D [15, 17–24].

The aim of the present study was therefore to evaluate if the two biomarkers together or separately could improve the predictive value for decline in renal function, incident CVD and all-cause mortality in T2D patients with microalbuminuria, preserved kidney function and no known cardiac disease.

## Materials and methods

### Participants and study procedure

In 2007–2008, we enrolled 200 patients with T2D, all Caucasians, from the outpatient clinic at Steno Diabetes Center Copenhagen, in an observational study. The purpose of this study was to investigate the prevalence and role of putative asymptomatic coronary artery disease in T2D patients with microalbuminuria and elevated plasma N-terminal-proBNP levels and/or coronary artery calcium score[25]. The same patients participated in the current prospective follow-up study. The inclusion criteria have been thoroughly described previously [25]. In brief, patients were eligible if they had T2D according to the WHO criteria, urine albumin excretion rate (UAER) > 30 mg/24h (two out of three consecutive measurements), normal kidney function and without any known cardiac disease. All participants received intensive multifactorial treatment constituting glycaemic, lipid and blood pressure (BP) control, antithrombotic therapy and lifestyle modification. The study complies with the Declaration of Helsinki, was approved by The Scientific Ethical Committee of the Capital Region of Copenhagen and all patients gave written informed consent.

### Baseline clinical and laboratory measures

Plasma concentrations of FGF-23 and GDF-15 were measured in all patients from samples collected in EDTA tubes and frozen at -80°C and stored in a research biobank for analysis immediately after the examination of the last participant. Thus, the samples were only thawed once and the maximal storage time of the samples prior to analysis was 13 months.

Plasma GDF-15 was measured using an immunoassay based on the electro-chemi-luminescence technology (Elecsys 2010)[26]. All measurements of GDF-15 were performed by Roche Diagnostics (Penzberg, Germany) by investigators blinded to the patient characteristics.

C-terminal FGF-23 concentrations were measured using a second generation enzyme-linked immunosorbent assay kit (Immunotopics, Inc, San Clemente, CA) with antibodies directed against two epitopes within the carboxyl-terminal proportion of FGF-23 molecule. All measurements of FGF-23 were performed at University of Miami (FL, USA).

UAER was measured in 24-h urine collections by an enzyme immunoassay (Vitros, Raritan, NJ, USA).

Current smoking was defined as one or more cigarettes, cigars or pipes a day. The eGFR was calculated applying the Chronic Kidney Disease Epidemiology Collaboration (CKD-EPI) equation[27]. Retinopathy status was assessed in all patients by fundus photography.

### Follow-up

The three predefined endpoints have previously been described [28, 29]. The combined CVD endpoint was defined as cardiovascular mortality, non-fatal myocardial infarction, stroke, ischaemic CVD and heart failure. For patients with multiple events, only the first endpoint was included. Annual measurements of p-creatinine were performed in 177 of the 200 patients as part of their regular diabetes control at SDCC. The renal endpoint was defined as a decline in eGFR>30% calculated from the p-creatinine at baseline to the latest available measurement, as proposed by Coresh et al.[30]. To increase power but also to address the issue of competing risk we also defined a composite renal endpoint (combining decline in eGFR>30% and all-cause mortality (as no cases of ESRD were seen).

Information on all patients was traced through the Danish National Death and the Danish National Health Registries on January 1, 2014. No patients were lost to follow-up. For deceased patients, information was obtained on the date and cause of death. All deaths were categorized as CVD unless an unambiguous non-CVD cause was present.

### Statistical analyses

Normally distributed variables are presented as means ±standard deviations (SD) and categorical variables as total numbers with corresponding percentages. FGF-23, GDF-15, UACR and 25(OH)-D vitamin were skewed distributed and log2 transformed in all analyses and summarised as medians with interquartile range (IQR). Clinical characteristics of the population at baseline were compared across the median concentrations of FGF-23 and GDF-15 by use of T-test for continuous and χ^2^ test for categorical variables.

Cox proportional hazards analysis was used to compute the hazard ratios (HR) with 95% confidence intervals (CI) per SD increment. A stepwise approach was applied: Model 1: unadjusted; Model 2: adjusted for sex, age and p-creatinine; Model 3: adjusted for: sex, age, LDL cholesterol, smoking, HbA_1c,_ p-creatinine, systolic BP and UACR. The analyses of FGF-23 were additionally adjusted for 25(OH)-D vitamin; and Model 4: Additionally inclusion of GDF-15 and FGF-23 mutually in the adjusted model.

We applied the Kaplan–Meier failure function to compare the risks of the renal endpoint of GDF-15 and of all-cause mortality according to the median levels of GDF-15 and FGF-23, respectively.

To evaluate the incremental contribution of FGF-23 and GDF-15 to standard risk prediction, we calculated the relative integrated discrimination improvement (rIDI), which has been suggested as a more powerful method to demonstrate the relative contribution of the new biomarker for improved risk prediction compared to standard approach [31].

A p-value of <0.05 was considered significant. Statistical analyses were performed using SAS software (version 9.4, SAS Institute, Cary, NC, USA).

## Results

### Patient characteristics

The population included 200 patients (76% men) with mean ± SD age of 59±9 years, eGFR 91.1 ± 18.3 ml/min/1.73m^2^ and median (IQR) UACR 103 (39–230) mg/24h. A total of 120 (60%) had retinopathy. The median FGF-23 and GDF-15 concentrations were 71 (52–108) relative units/ml and 1533 (1132–2236) ng/l, respectively. Clinical characteristics of the patients, categorized by the median of GDF-15 and FGF-23 respectively, are shown in Table 1. Patients with GDF-15 concentrations above the median were older, had longer diabetes duration, lower eGFR and LDL cholesterol and were more frequently treated with oral antidiabetic drugs than patients with GDF-15 concentrations below the median. Patients with FGF-23 concentrations above the median were more frequently men, had higher body mass index, lower eGFR and were more frequently treated with insulin compared to patients with FGF-23 concentrations below the median.

**Table 1:** Clinical characteristics of the study population at baseline categorized according to growth differentiation factor 15 and fibroblast growth factor 23 below or above the median.

	Growth differentiation factor 15 (μmol/l)		P	Fibroblast growth factor 23 (RU/ml)		P
	< 1533 (n = 100)	≥ 1533 (n = 100)		< 71.29 (n = 100)	≥ 71.29 (n = 100)	
Male, *n* (%)	73 (73)	78 (78)	0.41	84 (84)	67 (67)	0.005
Age (years)	55.7 ± 10.0	61.6 ± 2.9	<0.001	59.0 ± 8.8	58.3 ± 8.6	0.55
Known duration of diabetes (years)	14.1 ± 7.7	14.8 ± 7.6	0.012	12.3 ± 7.2	13.2 ± 7.5	0.39
Body mass index (kg/m^2^)	32.7 ± 5.8	32.4 ± 5.8	0.68	31.6 ± 5.0	33.5 ± 6.3	0.02
HbA_1c_ (%)	7.99 ± 1.44	7.74 ± 1.24	0.19	7.70 ± 1.25	8.02 ± 1.43	0.09
HbA1c (mmol/mol)	64±7.8	61±10	0.19	61±9.8	64±7.9	0.09
Urinary albumin excretion rate (mg/24-h)	116.5(43.0–207.5)	90.0(38.0–318.0)	0.79	107.0(44.0–189.5)	96.0(37.0–161.0)	0.71
P-creatinine (μmol/L)	70.2 ± 15.6	82.7 ± 18.8	<0.001	73.4 ± 16.9	79.5± 19.2	0.019
eGFR (ml/min/1.73m^2^)	96.5 ± 16.2	82.4 ± 15.5	<0.001	93.1 ± 16.3	85.9 ± 17.7	0.003
25(OH)-D vitamin (nmol/l)	37.1 (21.9–50.1)	36.7 (22.2–53.6)	0.75	37.9 (21.3–58.0)	34.3 (22.2–47.6)	0.52
LDL cholesterol (mmol/L)	1.99 ± 0.84	1.72 ± 0.69	0.015	1.83 ± 0.72	1.86 ± 0.83	0.89
Systolic blood pressure (mmHg)	132 ± 17	129 ± 18	0.29	131 ± 17	129 ± 17	0.50
Current smoker, *n* (%)	28 (28)	31 (31)	0.64	26 (26)	33 (33)	0.28
Retinopathy, *n* (%)	59 (59)	61 (61)	0.77	55 (55)	65 (65)	0.15
Treatment with:						
Oral antidiabetic, *n* (%)	79 (79)	91 (91)	0.018	83 (83)	87 (87)	0.43
Insulin, *n* (%)	60 (60)	64 (64)	0.56	52 (52)	72 (72)	0.004
Antihypertensive drugs, *n* (%)	99 (99)	99 (99)	1.00	99 (99)	99 (99)	1.00
Statin, *n* (%)	93 (93)	96 (96)	0.35	94 (94)	95 (95)	0.76
Aspirin, *n* (%)	91 (91)	92 (92)	0.80	91 (91)	92 (92)	0.80

eGFR: estimated glomerular filtration rate. P-values for differences between participants with Growth differentiation factor 15, and Fibroblast growth factor 23 below or above the median.

**Fig 1:**
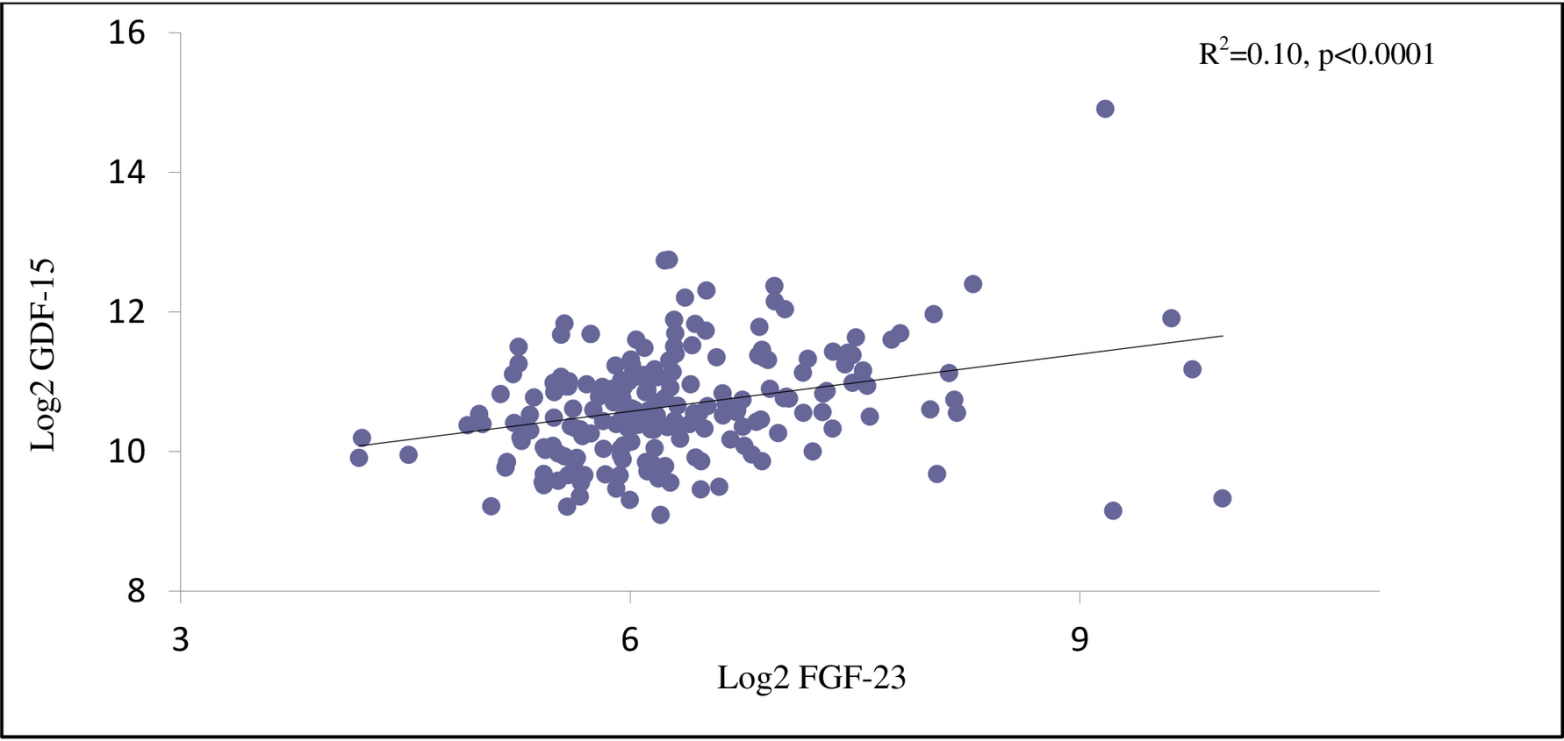
Scatterplot illustrating the significant correlation between log2-transformed GDF-15 and FGF-23 concentrations.

The vast majority of patients were receiving antihypertensive drugs (99%), statins (95%) and aspirin (90%). Fig 1 illustrates the positive correlation between GDF-15 and FGF-23 (R^2^ = 0.10; p<0.0001).

### Incidence of cardiovascular events, all-cause mortality and decline in eGFR

Median follow-up time was 6.1 (IQR: 5.9–6.6) years. During this period 40 patients experienced a combined CVD endpoint, 26 died and 42 had a decline in eGFR>30%.

The number of composite renal endpoint totalled 55.

The combined CVD endpoint included 11 fatal CVD events (2 cases of acute myocardial infarction, one case of ischaemic CVD, 6 sudden and otherwise unexplained deaths and 2 cases of heart failure) and 29 non-fatal CVD events (3 cases of acute myocardial infarction, 3 strokes, 19 cases of ischaemic CVD and four cases of heart failure). Of the 26 deaths, 11 were related to CVD, 9 cancer-related and 6 related to other causes.

The median follow-up for the renal endpoint was 4.9 (IQR: 3.8–5.4) years. In 23 patients the decline of eGFR>30% was confirmed in two or more measurements, whereas the decline in eGFR was observed at the last visit in 17 patients without the possibility of confirmation. None of the patients progressed to ESRD during follow-up.

### GDF-15 as a risk marker

Table 2 shows the associations of GDF-15 to the risk of fatal and non-fatal CVD events, all-cause mortality and decline in eGFR>30% per SD increment. In the unadjusted model higher GDF-15 concentration was a determinant of CVD events (p = 0.033), all-cause mortality (p = 0.003) and decline in eGFR>30% (p = 0.018), but only the relation to all-cause mortality (p = 0.003) and decline in eGFR (p = 0.018) kept significance after adjustment for traditional risk factors. Moreover, GDF-15 remained significant related to risk of all-cause mortality (p = 0.039) and decline in eGFR>30% (p = 0.013) after additional inclusion of FGF-23 in the model. Higher GDF-15 was also significantly related to the composite renal endpoint in all three adjusted models (p≤0.0019). The rIDI for GDF-15 was 30% (p = 0.037) for decline in eGFR>30% but was not significant for all-cause mortality (p = 0.077, Table 2).

**Table 2:** Stepwise Cox regression analyses: Biomarkers in relation to risk of fatal and nonfatal cardiovascular events, all-cause mortality and decline in eGFR >30% in 200 patients.

Biomarker	Model	Cardiovascular events (n = 40)	P	All-cause mortality (n = 26)	P	Decline in eGFR>30% (n = 42)	P
**GDF-15 log scale** **(1 SD = 0.55)**	Unadjusted	1.38 (1.03–1.86)	**0.033**	1.84 (1.32–2.57)	**0.0003**	1.75 (1.28–2.39)	**<0.001**
	Adjusted for sex, age and plasma creatinine	1.12 (0.78–1.63)	0.54	1.90 (1.31–2.77)	**0.0008**	1.65 (1.12–2.41)	**0.011**
	Adjusted*	1.25 (0.85–1.84)	0.25	1.90 (1.24–2.89)	**0.003**	1.67 (1.09–2.54)	**0.018**
	Adjusted* + FGF-23	1.18 (0.77–1.80)	0.44	1.64 (1.03–2.62)	**0.039**	1.73 (1.12–2.67)	**0.013**
**rIDI**				38.6%	**0.08**	30.0%	**0.04**
**FGF-23 log scale** **(1 SD = 0.28)**	Unadjusted	1.16 (0.86–1.56)	0.33	1.48 (1.10–2.00)	**0.010**	1.18 (0.88–1.59)	0.27
	Adjusted for sex, age and plasma creatinine	1.23 (0.90–1.68)	0.20	1.55 (1.14–2.10)	**0.0047**	1.05 (0.75–1.47)	0.77
	Adjusted*	1.16 (0.81–1.66)	0.41	1.57 (1.11–2.18)	**0.011**	0.98 (0.68–1.41)	0.90
	Adjusted*+ GDF-15	1.09 (0.74–1.60)	0.67	1.33 (0.89–1.99)	0.16	0.87 (0.59–1.28)	0.48
**rIDI**				29.7%	**0.02**		

Number of events given for each endpoint (n =).

Relative integrated discrimination improvement (rIDI): between a model including traditional cardiovascular risk factors only and a model including traditional risk factors and GDF-15 or FGF-23. GDF-15: growth differentiation factor 15, FGF-23: fibroblast growth factor 23, eGFR: estimated glomerular filtration rate. Values are hazard ratios with 95% confidence intervals, and represent an SD increment of log-transformed values of the biomarkers.

*Adjustment included sex, age, LDL cholesterol, smoking, HbA1c, plasma creatinine, systolic blood pressure and urinary albumin excretion rate. Analyses of fibroblast growth factor 23 was additionally adjusted for 25(OH)-D vitamin.

**Fig 2:**
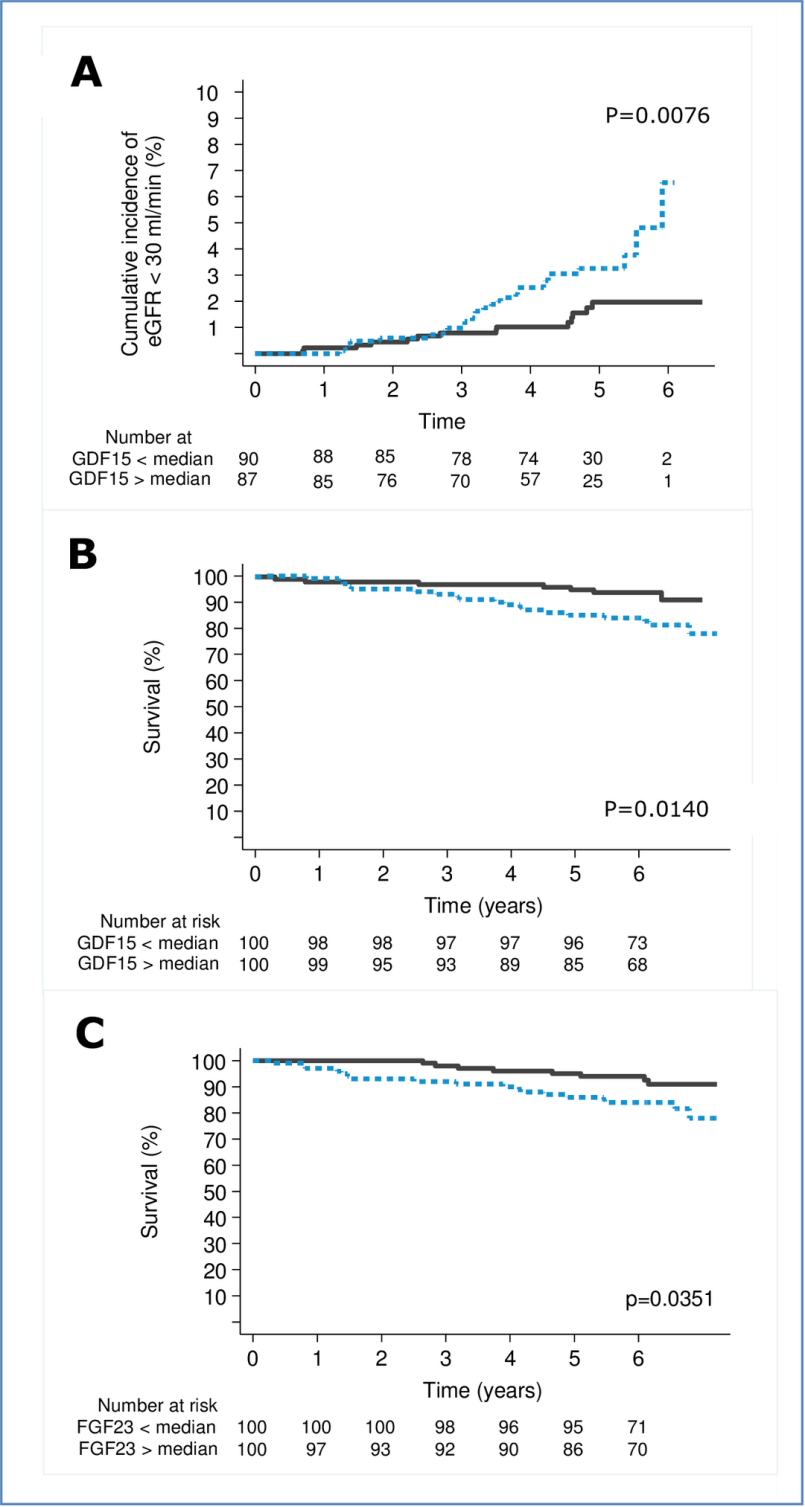
Kaplan-Meier plot of A. GDF-15 in relation to decline in eGFR>30%, B. GDF-15 in relation to all-cause mortality, C. FGF-23 in relation to all-cause mortality Numbers refer to participants in each category at risk at the beginning of each 2 year interval. P-values based on log-rank test.

The cumulative incidence of decline in eGFR>30% and all-cause mortality was higher in participants with GDF-15 above the median (p≤0.014; Fig 2, panel a and b) compared to participants with GDF-15 below the median.

### FGF-23 as a risk marker

Table 2 shows the associations of FGF-23 to the risk of fatal and non-fatal CVD events, all-cause mortality and decline in eGFR>30% per SD increment. Higher FGF-23 was not significantly related to CVD events or decline in eGFR, but was associated with all-cause mortality both in the unadjusted (p = 0.010) and adjusted (p = 0.011) model. After additional inclusion of GDF-15 in the model, FGF-23 lost significance (p = 0.16). There was not significant relation between FGF-23 and the composite renal endpoint (p≥0.31). The rIDI was 30% (p = 0.024) in relation to all-cause mortality (Table 2).

The cumulative incidence of all-cause mortality was higher in participants with FGF-23 above the median (p = 0.035; Fig 2, panel c) compared to participants with FGF-23 below the median.

## Discussion

In the present study of patients with T2D, higher GDF-15 independently predicted all-cause mortality and improved risk prediction of decline in renal function beyond traditional risk factors. Furthermore, higher FGF-23 independently predicted and improved risk prediction of all-cause mortality beyond traditional risk factors, but did not keep significance after inclusion of GDF-15 in the model.

### GDF-15 as a risk marker

GDF-15 is expressed in various tissues, cells and organs with p53 protein as the direct molecular target, induced by tissue injury, anoxia and pro-inflammatory cytokines responses[32]. In general, GDF-15 plays a pivotal role in the development and progression of CVD [33]. In our study of microalbuminuric T2D patients, higher GDF-15 was associated with CVD (event rate 20%) in unadjusted analyses, but did not predict CVD after adjustment for traditional risk factors, and in addition predicted all-cause mortality after additional adjustment for FGF-23. In a study including 861 patients with T2D and advanced nephropathy (mean eGFR 33 ml/min/1.73m^2^), no association was found between GDF-15 and the composite CV endpoint including CV mortality (event rate 12.4%) after adjustment for traditional risk factors comparable to our adjustment[18]. Yet, in another study including 213 T2D patients without albuminuria, CKD or CVD, higher GDF-15 did predict CVD in terms of diabetic cardiomyopathy (event rate 21.1%), but adjustment did not include important risk factors such as BP, lipids, creatinine and UACR [34]. However, in another study in 746 T2D patients also without albuminuria and CKD, Resl et al. found an association between higher GDF-15 and CVD (event rate 22.9%) even after adjustment for a comprehensive panel of risk factors similar to our adjustment [20, 35]. In our previous study in 451 patients with T1D and macroalbuminuria (measured mean GFR 76±33 ml/min/1.72m^2^), higher levels of GDF-15 predicted CVD and all-cause mortality. This could not be confirmed in the 440 normoalbuminuric T1D patients, possibly due to few CVD events (n = 48) [26]. In summary, these studies in T1D and T2D show conflicting results which partly might be due to differences in kidney function, event rates and adjustments.

Recently, Nair et al showed higher circulating GDF-15 concentration to be positively correlated with intrarenal expression of GDF-15 and an increased risk of CKD progression in two independent non-diabetic cohorts with CKD[19]. This indicates a more direct role of GDF-15 in the renal disease process. In our previous as well as present studies in T1D[26] and T2D respectively, higher GDF-15 predicted decline in renal function beyond traditional risk factors. This is in accordance with a study of T2D patients with normal renal function, where higher GDF-15 predicted renal outcome in terms of progression of albuminuria[17]. In T2D patients with CKD, the renal endpoint including doubling of baseline serum creatinine level, incident ESRD or a sustained 40% decline in eGFR could also be predicted by higher GDF-15[18]. Thus, these studies suggest that higher GDF-15 is an independent risk marker for renal disease in T2D from early to late stages of dysfunction.

### FGF-23 as a risk marker

FGF-23 is predominantly secreted by osteocytes. It regulates the phosphate homeostasis by inhibiting the sodium dependent phosphate transporter and thus phosphate reabsorption in the proximal tubule, hence increasing phosphate excretion. Likewise FGF-23 inhibits 1α-hydroxylase and thereby the synthesis of 1.25-dihydroxy vitamin D leading to decreased absorption of phosphate from the gut. Higher FGF-23 is perceived as one of the earliest markers of kidney disease, reaching very high levels at the time of ESRD[36]. Despite these apparently beneficial physiological effects, increased concentrations of FGF-23 have been linked to CV events, mortality and initiation of dialysis[7, 8, 10–14]. In our study, higher FGF-23 predicted all-cause mortality, but did not relate to CVD or decline in renal function. In a previous analysis of the same population, with a shorter follow-up period (median 3.5 years), we could not demonstrate any relation either between FGF-23 and yearly decline in eGFR or progression in albuminuria[24]. Even though the study population had some degree of kidney involvement in terms of elevated albumin excretion, the kidney function was normal and median FGF-23 concentration was within the normal range. In contrast, a large community based study also with FGF-23 concentration within normal range followed for 19 years, demonstrated an association between higher FGF-23 and increased risk of incident ESRD independent of kidney function[8]. In a mixed diabetic and non-diabetic cohort of patients with coronary heart disease, higher FGF-23 predicted CV outcomes in the diabetic but not in the non-diabetic patients suggesting potential biological differences in the FGF-23 response between patients with and without diabetes[23].

In T2D, two studies in patients with CKD, higher FGF-23 predicted a decline in renal function[15, 21]. In another study in T2D, where the majority had normal renal function and 51% had normoalbuminuria, higher FGF-23 was, like our results, independently associated with increased risk of all-cause mortality, but was, unlike our findings, also associated to CV outcome and risk of ESRD [22]. Several factors might explain the observed differences. The population included various degrees of diabetic renal disease, where our cohort had more homogenous renal status, the follow-up time was longer (8–12 years), 13% of the events was ESRD (n = 48), with no ESRD events in our cohort, and eGFR was calculated based on the MDRD rather than the CKD-EPI equation, as in our study.

### Strength and limitations

The well-defined phenotype of the cohort and no patients lost to follow-up is a strength. The relative small amount of renal events is a limitation. There are baseline differences regarding important risk factors for kidney function and mortality. This renders the possibility of residual confounding, despite correction for these parameters. When investigating development of decline in renal function, all-cause mortality might be a competing risk. To address this issue, we defined a composite renal endpoint including decline in renal function and all-cause mortality. We did not have information on the use of vitamin D or calcium supplements, or measures of phosphate, parathyroid hormone or calcium levels, which might potentially interact on the FGF-23 concentration[36]. It is however most likely, that our population, due to the normal kidney function, had normal concentrations of phosphate, parathyroid hormone and calcium. The definition of kidney function was based upon estimated GFR rather than a more precise measure[37], such as 51-Chrome-EDTA clearance. The majority of the patients had diabetic retinopathy indicating that the albuminuria most likely was due to diabetic kidney disease. However, 40% did not have retinopathy and as diagnostic renal biopsies was not performed; the presence of other non-diabetic renal disease cannot be ruled out.

### Clinical implications

Higher GDF-15 remained a significant predictor of all-cause mortality and renal decline after additional inclusion of FGF-23 in the adjusted model indicating thatGDF-15 is a stronger risk marker than FGF-23 for these endpoints. This might suggest that there is no added value of FGF-23 when GDF-15 is measured. However the mechanisms’ underlying the associations between FGF-23 and GDF-15 on one side and mortality and renal disease on the other remains unclear. Whether higher concentrations of FGF-23 have a detrimental effect on the vasculature and kidneys or simply acts as a surrogate marker for presence of cardiac or renal injury is unsolved. In order to reduce FGF-23 concentrations, interventions should potentially block or reduce phosphate absorption pharmacologically and/or through reduced dietary phosphate intake. On a more experimental level biological agents directly targeting or blocking the actions of FGF-23 in terms of inhibitory anti-FGF-23 antibodies are in development but without convincing results[38]. Likewise approaches to lower GDF-15, like treatment with antioxidant or anti-inflammatory agents, may be associated with improved outcome[16]. Prospective randomized studies of such interventions are needed to clarify if reductions of the biomarker can translated into improved outcomes before consolidating these markers.

## Conclusion

In microalbuminuric T2D patients with normal kidney function and without known cardiac disease, higher concentration of GDF-15 predicted decline in renal function, CV events and all-cause mortality. After adjustment for traditional risk factors, the prediction for decline in renal function and all-cause mortality persisted. Higher FGF-23 concentration was an independent risk marker for all-cause mortality, but was no longer significant after inclusion of GDF-15 in the model. Higher GDF-15 was a risk marker for the renal endpoint and all-cause mortality independently of the level of FGF-23.
